# Delineating natural catchment health districts with routinely collected health data from women’s travel to give birth in Ghana

**DOI:** 10.1186/s12913-022-08125-9

**Published:** 2022-06-13

**Authors:** Winfred Dotse-Gborgbortsi, Andrew J. Tatem, Zoë Matthews, Victor Alegana, Anthony Ofosu, Jim Wright

**Affiliations:** 1 School of Geography and Environmental Science, University of Southampton, Southampton, S017 1BJ UK; 2 WorldPop, School of Geography and Environmental Science, University of Southampton, Southampton, UK; 3 Department of Social Statistics and Demography, University of Southampton, Southampton, UK; 4 Population Health Unit, Kenya Medical Research Institute – Wellcome Trust Research Programme, Nairobi, Kenya; 5 Ghana Health Service, Headquarters, Accra, Ghana

**Keywords:** Health catchment areas, Maternal health services, Health information systems, Geographic information systems, Health systems plans, Health services accessibility

## Abstract

**Background:**

Health service areas are essential for planning, policy and managing public health interventions. In this study, we delineate health service areas from routinely collected health data as a robust geographic basis for presenting access to maternal care indicators.

**Methods:**

A zone design algorithm was adapted to delineate health service areas through a cross-sectional, ecological study design. Health sub-districts were merged into health service areas such that patient flows across boundaries were minimised. Delineated zones and existing administrative boundaries were used to provide estimates of access to maternal health services. We analysed secondary data comprising routinely collected health records from 32,921 women attending 27 hospitals to give birth, spatial demographic data, a service provision assessment on the quality of maternal healthcare and health sub-district boundaries from Eastern Region, Ghana.

**Results:**

Clear patterns of cross border movement to give birth emerged from the analysis, but more women originated closer to the hospitals. After merging the 250 sub-districts in 33 districts, 11 health service areas were created. The minimum percent of internal flows of women giving birth within any health service area was 97.4%. Because the newly delineated boundaries are more “natural” and sensitive to observed flow patterns, when we calculated areal indicator estimates, they showed a marked improvement over the existing administrative boundaries, with the inclusion of a hospital in every health service area.

**Conclusion:**

Health planning can be improved by using routine health data to delineate natural catchment health districts. In addition, data-driven geographic boundaries derived from public health events will improve areal health indicator estimates, planning and interventions.

## Background

Demarcating geographic areas from patient travel is essential for health policy, planning, and research [1]. Health service managers use spatial tools to plan health services, identify gaps in service provision, prioritise areas with poor access, measure service coverage and allocate resources [2]. In some countries, such as Uganda and Ghana, district league tables based on these boundaries have formed the basis for health systems performance assessment [3, 4]. Health catchments can be natural, reflecting patient choice, or mandated, reflecting regulation of health service delivery [5]. For instance, general practices follow mandated catchments in the UK because patients must live within practice boundaries [6]. However, some countries, including Ghana, have natural catchments because patients can use any health facility. While supply is fixed within a district, significant flows of cross-border service users can change the demand for services [7] and affect management priorities and planning needs. Identifying catchments is vital for measuring coverage, access, improving quality and planning [8–10]. Therefore, zone design approaches can help by sub-dividing large regions or merging small areas into health service areas, or even changing boundaries altogether.

A health service area (HSA) is a geographic unit where most of the trips to use routine health services start and end internally within that geographic unit, making it relatively self-contained [11]. HSAs improve quality of care and provide insights into service utilisation patterns and access to care by supporting areal health indicator reports [8–10, 12, 13]. Additionally, health service area demarcations can be used for health systems programming. However, planning health services within district boundaries can be problematic, particularly in districts with overutilised hospitals or without secondary care facilities.

The Travel-To-Work-Area algorithm (TTWA) is one of the approaches for delineating zones [14]. The TTWA has been used to delineate labour market [14], retail [15] and health service areas [16, 17]. It was applied to admissions to develop health service areas [17] and general practice affiliations data for local health planning purposes [16]. The TTWA is useful for zone design because it creates non-overlapping zones by maximising internal flows and minimising flows between zones.

For birthing services, reaching health facilities promptly in times of need and emergency is crucial for promoting women’s health, and preventing maternal mortality and disability [18]. Furthermore, a functioning referral system includes effective communication between service providers to guarantee timely quality care [19]. Inadequate coordination and communication between health facilities during referrals can lead to adverse outcomes for women and their babies [20, 21]. Health professionals within a hospital network need knowledge of the nearest available quality secondary care to prepare for life-saving emergencies adequately. Information on available resources can be shared between collaborating health facilities within a HSA.

Access to maternal health service indicators can be calculated using HSA demarcations [9]. For instance, the World Health Organisation (WHO) recommends five (four basic and one comprehensive) emergency obstetric care facilities per half a million population within a geographical area [22]. In terms of staff based at these crucial health facilities, at least 22.8 skilled health workers (midwives, nurses, doctors) per 10,000 population threshold are recommended. This benchmark has more recently been revised upwards to 33.45 skilled health workers per 10,000 population [23]. A set of key suggested indicators measuring performance against staffing benchmarks, facility benchmarks, health outcome measures, and quality markers can be estimated using the HSA and administrative boundaries. Therefore, geographic boundaries play a fundamental role in robust reporting of areal statistics to improve maternal health quality.

The problem with using government administrative boundaries to measure health indicators such as the physician-to-population ratio is the magnitude of cross-district use of health services [9]. In Ghana, health planning and other public health management activities are limited to government administrative boundaries. However, service use goes across district boundaries as clients move from one district to another for services [24]. Therefore, midwife-to-population ratios and other maternal health access indicators could misrepresent the demand for services.

Almost all studies delineating HSAs using admissions data are in high-income countries [8, 9, 13, 25]. Meanwhile, the increasing availability of individual-level routine health data in low-income settings with electronic health systems can aid such studies [26]. Although some studies have estimated catchment areas around individual health facilities for malaria and other infectious diseases, these statistical methods are not optimised for zoning HSAs because the catchments are drawn around individual health facilities [27–29]. Also, they focus on travel times and care-seeking behaviour rather than developing HSAs for reporting health service performance indicators. However, evidence from high-income countries suggests that contiguous zones formed by grouping health facilities using patient flows are well suited for healthcare planning [25]. Despite the advantages of this approach, no existing studies demarcated HSAs in low and middle income countries (LMIC) using patient flows. A study in Ghana divided one district into sub-districts with network analysis [30].

This study aims to delineate HSAs using routine birth admission data. Also, the study examines to what extent the TTWA method can be adapted to design zones using patient flows to secondary health care facilities. In doing so, we merge highly connected health facility zones to form HSAs. Subsequently, the creation of HSAs allows this study to estimate maternal health indicators for planning and to improve maternal health services within the study area.

## Methods

### Data

Data documenting births of 32,921 women from 27 hospitals in the Eastern region, Ghana, from 1st January to 31st December 2017 were used to analyse women’s travel to give birth. The Ghana Health Service (GHS) collects birth data using book registers. Midwives record details of a woman into these registers when they give birth in a health facility. Information collected includes the woman’s residential address, age, parity, complications, birth outcomes and other relevant maternal health information.

Subsequently, the data is entered into the electronic District Health Information Management System (DHIMS). First, the women are counted and reported as monthly aggregates. Secondly, the individual records as they appear in the register are also captured in the DHIMS system. Currently, only hospitals enter the individual women’s data into the DHIMS, as health centres use paper registers only. The individual records transferred from paper to electronic registers at hospitals can differ from routine aggregate reports. The two key variables used in this analysis are the woman’s community of residence and the health facility she gave birth in.

District and sub-district boundaries from the GHS were included in the analysis. In the GHS, sub-districts are the lowest administrative areal unit and are formed by a group of health facilities and communities. We used WorldPop gridded (100 m by 100 m) estimated population, the number of midwives in health facilities, and the spatial distribution of the hospitals to estimate access to adequately staffed birthing services. The WorldPop group produces the population estimates by disaggregating census data into 100 square meter grids within built settlements using Random Forest machine learning methods [31].

In order to construct indicators that provide a good measure of human resource availability and quality of care, data were collected for this study in September 2021. An emergency obstetric and newborn care (EmONC) service provision assessment survey (SPA) was conducted. The survey data was used to determine if hospitals provided care to the level of Comprehensive Emergency Obstetric and Newborn Care (CEmONC). A hospital is classified as CEmONC ready if they administered parenteral antibiotics, uterotonics, parenteral anticonvulsants, removed placenta manually, removed retained products, performed assisted vaginal delivery, neonatal resuscitation, caesarean section and blood transfusion in the last 3 months [22]. CEmONC designated health facilities are supposed to be ready for all major obstetric complications, including the need for surgery and blood transfusion.

### Birthing mobility patterns

We used a list of place names with geographic coordinates from the GHS to locate the residential towns of the women. However, we could not find some addresses due to spelling errors, unavailable town names or address mismatches. The town names were manually matched with the reference list as automated geocoding performs poorly because official standardised address lists are unavailable in Ghana [32]. The geographic locations of the hospitals were collected during the SPA.

The flows of women initially captured between residential communities and hospitals were aggregated to sub-districts. The aggregation was carried out for two reasons: to reduce the complexity of flows and, secondly, to be consistent with the geographic unit used for delineating HSAs in subsequent analysis. For mappings, only flows of six or more women between sub-districts were shown to avoid graphical complexity [33].

### Zoning health service areas

**Fig. 1:**
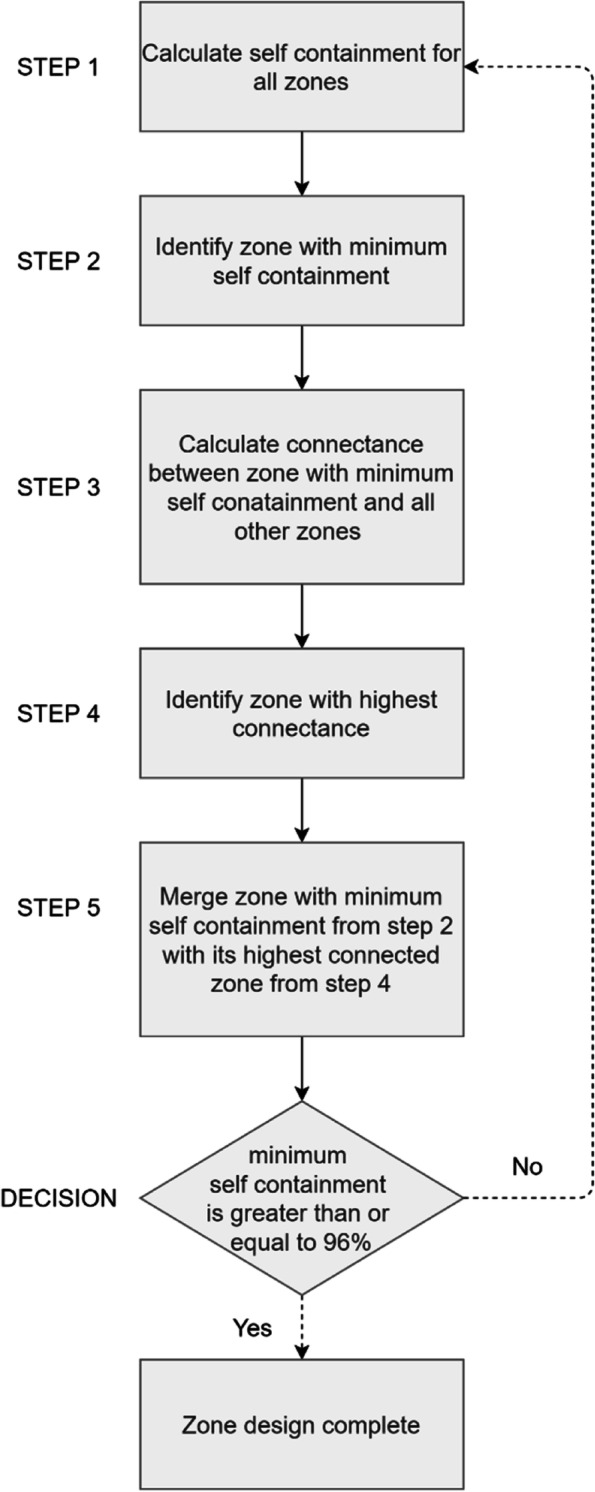
Zoning procedure used to delineate natural catchment health districts in Eastern Region, Ghana

The TTWA zoning method used in this study is a criteria-based zoning process originally used to delineate labour market [14] and retail [15] areas from flow data. The TTWA was used to analyse labour markets delineated to have the majority of people living and working within the zones generated. Similarly, this study utilises it to create health service areas where people live and use birthing services in an area. The building block for our zoning analysis is sub-districts, which were merged into larger areal units. Sub-districts without any facilities reporting births were first merged to a nearby one within the same district. Figure 1 shows the steps involved in developing the HSAs, implemented via Visual Basic within a Microsoft Access database.

The women were first assigned to the hospital they used. Where women in a subdistrict used more than one hospital, they were assigned to the hospital that received the most flows from that subdistrict to form the first set of zones. Then, the self-containment or localisation index was calculated (Step 1) to determine which zone will be merged in the next step. Next, demand and supply-side self-containment are calculated from patient flows (Step 2). Demand-side self-containment is calculated as the number of internal flows starting and ending within a zone as a proportion of all flows ending in the zone. In contrast, supply-side self-containment comprises internal flows as a proportion of all flows originating in a zone. The zone with the minimum demand or supply-side self-containment is the candidate for merging.

The next step (Step 3) calculates the connectance (connectivity strength) between zones [15] to identify the zone with the highest connectance (Step 4). The connectance flows are calculated between the zone with the lowest self-containment in step two and all other zones. In Step 5, we merged the least self-contained zone in Step 2 with the best connected in Step 4. This analysis used the connectance flow function (Eq. 1) by Pratt and colleagues [15] derived from Coombes [34]:

1 $${C}_{ij}=\left(\frac{T_{ij}}{\sum_i{T}_{ij}}\ x\ \frac{T_{ij}}{\sum_j{T}_{ij}}\right)+\left(\frac{T_{ji}}{\sum_i{T}_{ji}}\ x\ \frac{T_{ji}}{\sum_j{T}_{ji}}\right)$$

Where *C*_*ij*_ is the connectance flows between zone *i* and zone *j.*

*T*_*ij*_ are the flows from zone *i* to zone *j.*

*T*_*ji*_ are the opposite from zone *j* to zone *i.*

The sum of flows at the origin ∑_*i*_*T*_*ij*_ contains internal flows from *i* to *i*.

The sum of flows at the destination ∑_*j*_*T*_*ij*_ contains internal flows from *j* to *j*.

The sum of reverse flows at the origin ∑_*i*_*T*_*ji*_ contains internal flows from *i* to *i*.

The sum of reverse flows at the destination ∑_*j*_*T*_*ji*_ includes internal flows from *j* to *j*.

Steps one to five are repeated until a minimum self-containment criterion is met. Zones were only progressively merged. They were not dissolved as implemented in the original TTWA process.

The minimum supply/demand self-containment threshold for all zones to qualify as an HSA was set at 96%. The self-containment was high because the initial self-containment was high (70.5%), and a high value optimises health services planning by limiting cross border patient movement in the output zones.

The final step used manual interventions to make all zones contiguous. There were instances where most of the women in a sub-district attended the regional hospital or a hospital farther away, resulting in an outlier island zone. These inconsistent zones were corrected by assigning them to the nearby zone with the highest contiguity to ensure homogeneity.

Henceforth, the study introduces new terminology and refers to HSAs as Natural Catchment Health Districts (NCHD) as it differentiates them from HSAs delineated from mandatory catchments. NCHD and Zones are used interchangeably.

A simpler comparable set of zones were delineated to assess the effect of scale on self-containment. The flows were assigned to the destinations where most women went to give birth from a sub-district. The zone with the smallest supply-side self-containment was a candidate for merging. The candidate zone was merged to the contiguous zone with the least supply-side self-containment. The process was repeated until a comparable number of zones was achieved.

### Health planning indicators

Geographic access indicators were calculated using municipal and district assembly boundaries (MDA) and NCHD. MDAs are the government geographical boundaries for local governance. Two indicators of access to birthing services were calculated for each geographic boundary using the 2021 service provision assessment survey and gridded population data:

1. The number of CEmONC hospitals per 100,000 population
2. The number of midwives per 10,000 population

Although the most appropriate indicators of geographic access are travel time to health facilities [5], the analysis used a provider to population ratio. Provider to population ratio is suitable because it is the primary indicator health managers use, simple to calculate and a recommended benchmark by the WHO [23, 35].

## Results

### Women’s journeys to give birth in secondary care facilities

**Fig. 2:**
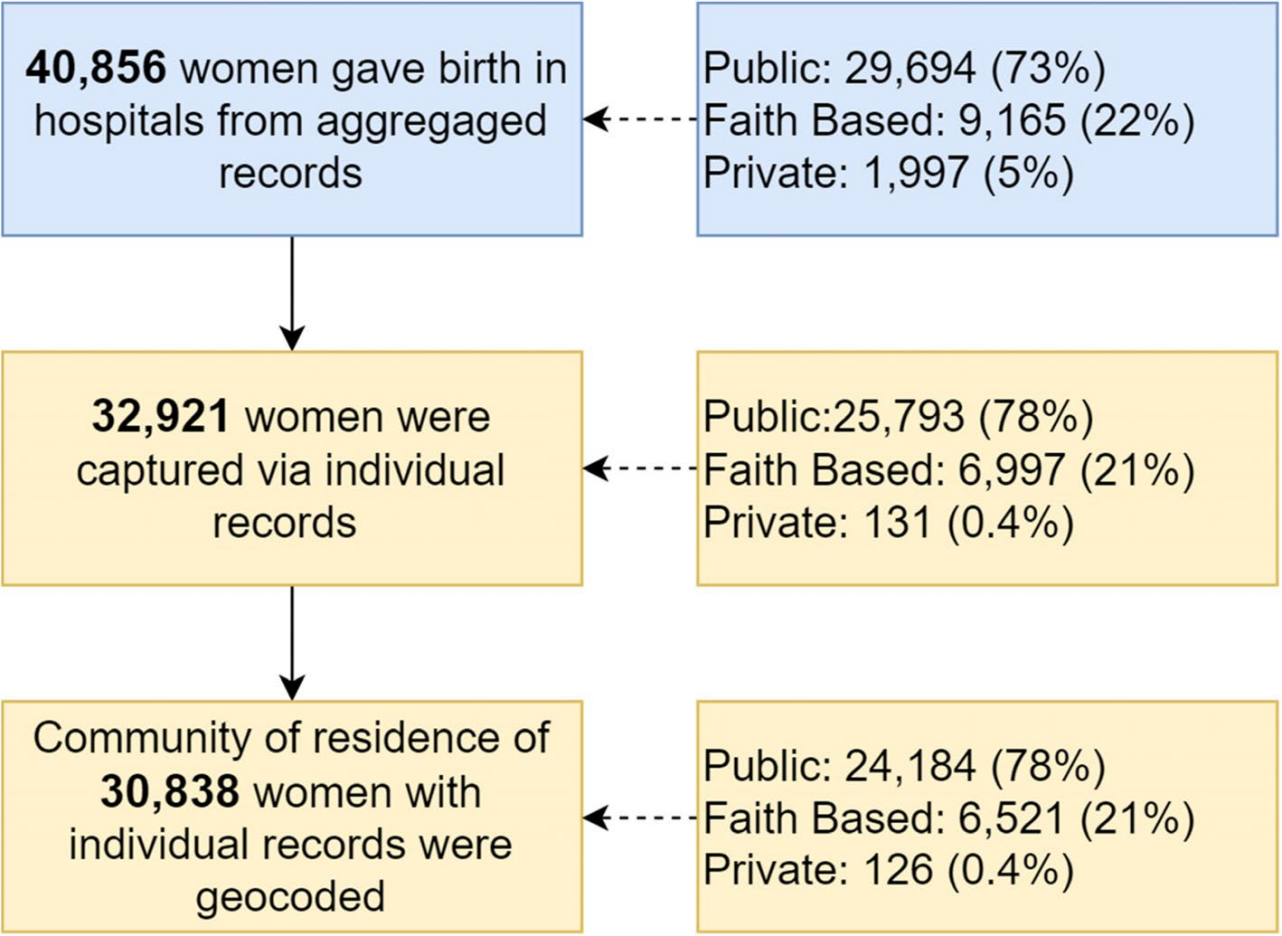
Participant flow diagram showing data completeness and the proportion of data by health facility sector (public, faith-based and private)

There were 32,921 (80.6%) individual birth records of the 40,856 aggregated counts in the DHIMS database (Fig. 2). Geographic coordinates of the residential communities were successfully identified for 30,838 (93.7%) of women with individual records. However, there was no substantial variation in the proportion of records by facility ownership (government, private, faith-based) and availability of geographic coordinates. The 30,838 women analysed came from 1015 residential communities in 250 sub-districts and gave birth in 27 hospitals. The number of women making journeys from a residential community to a hospital pair ranged from one to 1726.

**Fig. 3:**
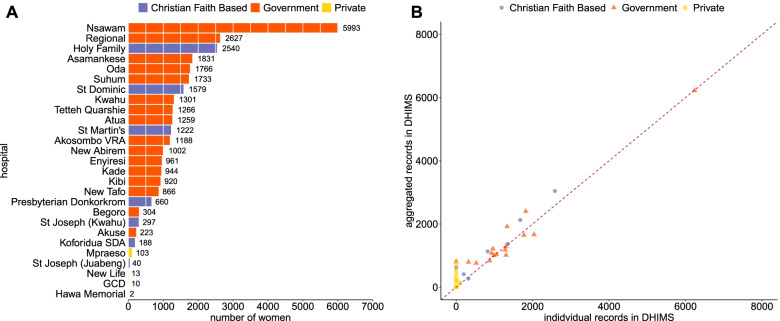
**A** The number of women giving birth by hospitals in Eastern Region, Ghana, 2017, based on individual-level birth register records. **B** Individual versus aggregate records reported in DHIMS in Eastern Region, Ghana, 2017; the red line shows expected 100% correlation between the individual and aggregated data

On average, there were less than 30 births per residential community in 2017 and a median of 7 births [Inter Quartile Range IQR = 20, (2 to 22)], but this skewed distribution has a few communities with many more births. The total births at the top 50 residential communities ranged from 115 to 2002. Private and faith-based hospitals saw fewer women than public hospitals (Fig. 3A). Inter-regional users from the Greater Accra area (women who crossed regional boundaries from the national capital city) represented more than one quarter (26%) of women from the top 10 residential communities. The agreement between aggregated and individual reports are compared in Fig. 3B. There was a 97.1% correlation between the aggregated and individual reports in DHIMS. Non-reporting hospitals were mainly private.

**Fig. 4:**
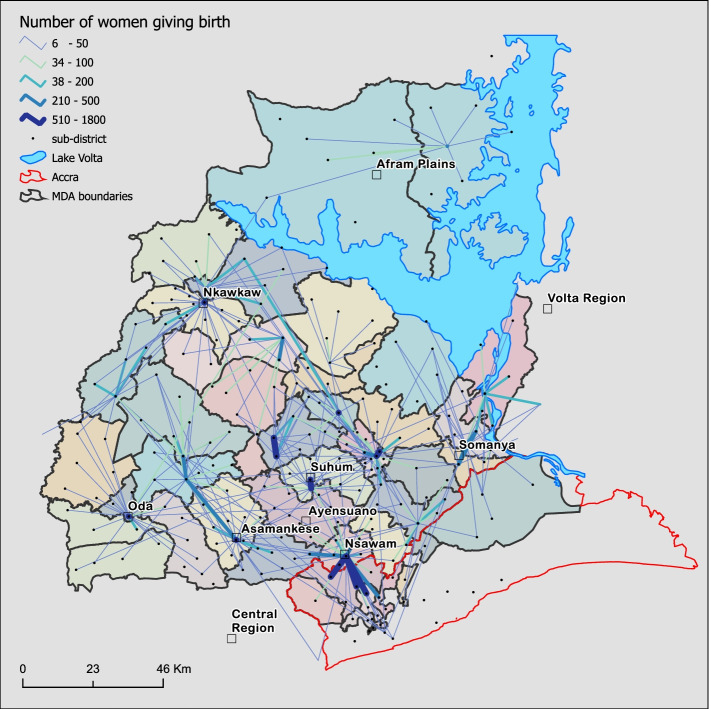
Flow pattern of 30,838 women giving birth in Eastern Region, Ghana, 2017. Flows are shown from residential sub-district to hospitals. Larger line width and deeper colour shading denote a larger number of flows

Women moved between and within MDAs to give birth (Fig. 4). The number of women travelling to give birth decreased with increasing distance to hospitals as larger flows lived closer to the hospitals. Afram plains areas, surrounded by the lake, had a highly localised movement pattern. Women from the Central, Volta and Greater Accra regions used nearby hospitals in the Eastern Region. However, the most substantial inter-regional flows were women from Greater Accra, primarily using Nsawam hospital. Almost half [2894 (48%)] of the women giving birth at Nsawam hospital were from Accra.

### Delineation of natural catchment health districts

**Fig. 5:**
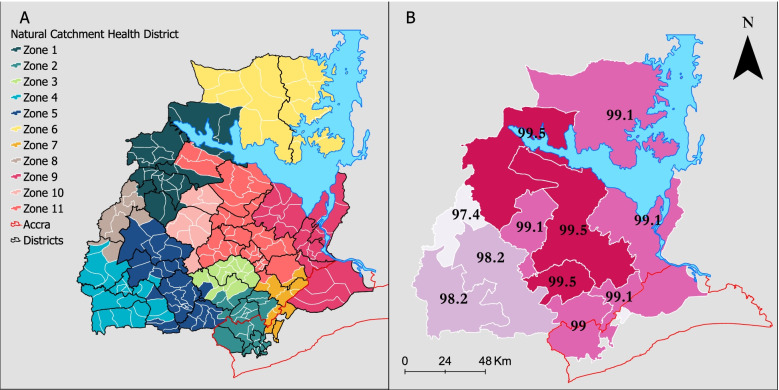
**A** NCHD derived from the flows of women giving birth in Eastern Region, Ghana, 2017, based on individual-level birth register records. **B** Minimum self-containment (supply or demand) in each zone indicates the compactness of flows within NCHDs

**Fig. 6:**
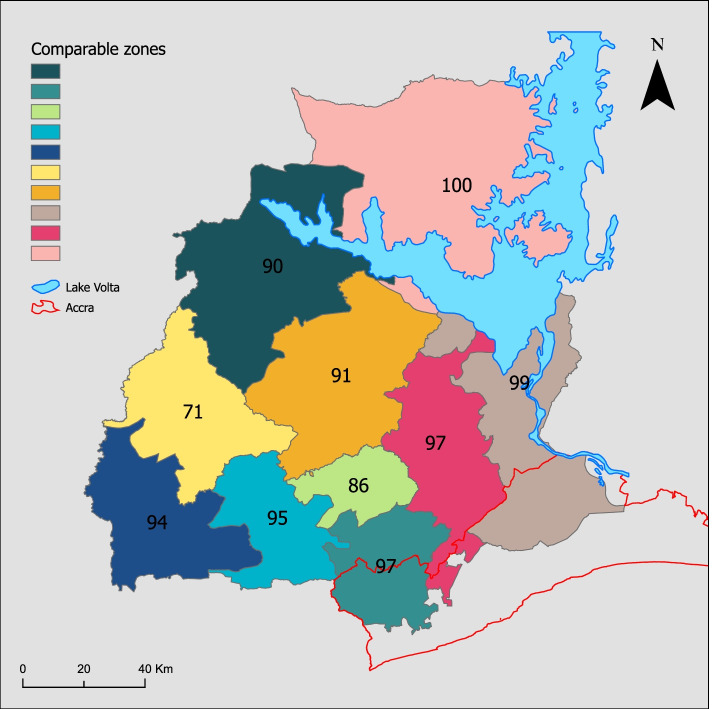
Comparable zones delineated to evaluate the effect of scale on the NCHD. Each zone is labelled with their self-containment

Based on the women’s flow patterns and connectivity between the hospitals, 11 natural catchment health districts (NCHD) emerged from the 250 sub-districts (Fig. 5A). The zones vary from 473 km^2^ (zone 7) to 4669 km^2^ (zone 6). The Afram plains area (zone 6), surrounded by Lake Volta, was highly self-contained without connecting to other zones through all iterations. Each NCHD covered at least two or more districts. There were an average of six administrative districts in each NCHD. The only district split between three zones (Ayensuano) does not have a hospital. The final self-containment was high, with 97.4% to 99.% of journeys starting and ending within the output zones (Fig. 5B). The merging of zones in each iteration increased the self-containment. Similarly, the difference between the internal flows for all output zones and each output zone decreased anytime zones were merged. The self-containment for the comparable zones (Fig. 6) ranged from 71 to 100% for each output zone and 91% altogether.

### Access to birthing service indicators by MDA versus NCHD

**Fig. 7:**
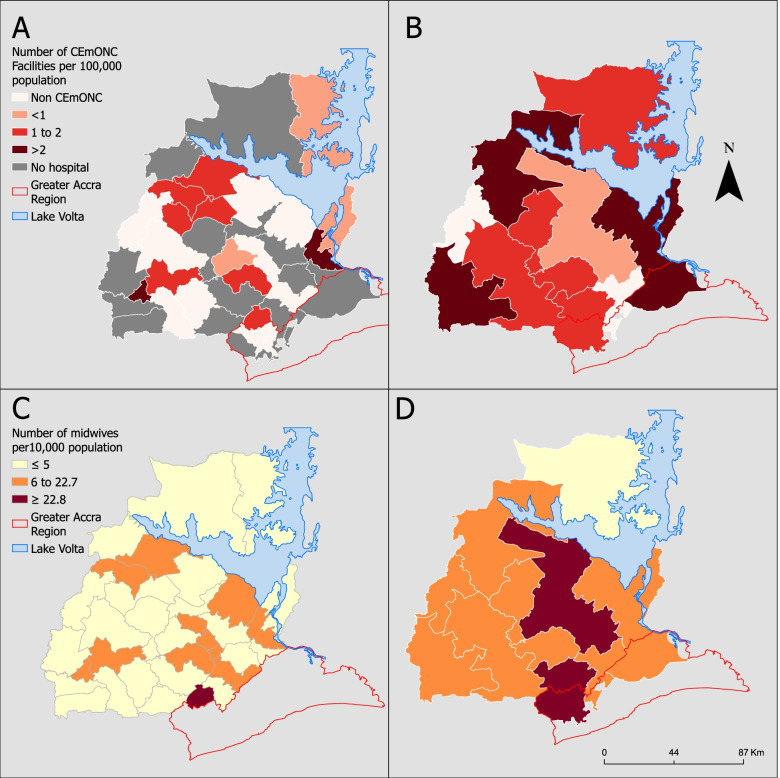
Access to hospitals ready to provide CEmONC services per 100,000 estimated pregnancies in **A** MDAs, **B** NCHD. The midwife-to-population ratio per 10,000 population in **C** MDAs, **D** NCHD

Figure 7 shows the geographical distribution of hospitals providing CEmONC services and the midwife-population ratio. Of the 33 MDA areas in the Eastern region, 12 (36%) did not have a hospital, and 10 districts (30%) had hospitals that were not ready to provide CEmONC (Fig. 7A). Most of the remaining 11 districts either had one or two CEmONC hospitals per 100,000 population. When zoned, all NCHDs had hospitals, but two were without CEmONC (Fig. 7B).

There were marginal variations between the number of hospitals and midwives attending to pregnancies across the region. Although there was only one public hospital in some districts (e.g. Nkawkaw), the number of midwives was high (Fig. 7C). There were two or more hospitals in two districts. There were an average of five and 15 midwives per 10,000 population in MDA and NCHD, respectively. Only one district and three NCHDs meet the 22.8 per 10,000 population WHO threshold.

## Discussion

This study presents the first analysis delineating NCHD using birth data from routine health records. Likewise, it provides the first insights into delineating NCHDs from flow data in Ghana and any LMIC setting. The study merged catchments of 27 hospitals in 33 administrative districts to 11 zones using birthing records. The minimum proportion of internal flows of women giving birth within these zones was 97.4%. Accra’s interregional flows to the Eastern region were prominent in all districts sharing a border with Accra, especially Nsawam. This study introduced a new term, NCHD, specific to health service areas or zones delineated from natural flow patterns.

### Implications for maternal health planning and policy

In Ghana and many other LMICs, district boundaries are used across all government sectors such as agriculture, education, and local government. While this MDA structure harmonises boundaries for all government sectors and fosters cross-sectoral cohesion at the district level, they are not optimised for any particular government sector. Indeed, population size and political considerations have played the most part in shaping district boundaries [36]. There are mismatches between the MDAs and the distributions of hospitals because not every MDA boundary contains a district hospital. There are similarly no hospitals within some second-level administrative boundaries elsewhere in Ghana and Sub-Saharan Africa [37]. Furthermore, since the MDAs are not designed to capture a group of health facilities and the population attending those facilities, they are problematic as a basis for areal health indicators.

The NCHDs address the mismatched distribution of hospitals versus MDAs. First, the NCHDs impact the calculation of indicators and positively address mismatch challenges associated with MDAs by providing a more robust basis for calculating indicators. The high self-containment in NCHDs ensures that areal health indicators reflect the attending population. Likewise, indicators such as mortality rates, caesarean sections, skilled birth rates and other estimates are reasonable because every NCHD has a hospital. The results reveal NCHDs with inadequate human resources and poor emergency obstetric care. When these quality care and human resource challenges are addressed, they could improve the skilled birth coverage in Ghana [38, 39].

Topography likely influences movement patterns and NCHDs. For instance, the Afram plains area surrounded by the Volta lake had no connectivity with other districts, as did the mountainous Akwapim North with its unsurfaced roads in some communities, forming one zone. The influence of topography on access to care should be considered when upgrading hospitals or improving the quality of maternal health. If service quality in these hospitals is inadequate, women would struggle to access alternative, higher quality hospitals. They might give birth at home since poor roads and the inability to cross rivers are risk factors for home births in rural Ghana [40].

The NCHD zones may be helpful in the organisation of health professional teams. High connectance values are likely to reflect patient choice and referral patterns. For example, hospitals within the same NCHD are likely to be referring women between neighbouring hospitals in emergency cases. Maternal health quality improvement projects based on collaborating health teams increased skilled birth uptake in Ghana [41]. Thus, if health professionals were geographically grouped into teams within the zones, this might further improve referrals and promote continuity of care. A new GHS “network of practice” initiative could also benefit from our zoning approach to group health facilities [42]. The initiative aims to group health facilities to collaborate, share resources, improve referrals, and provide technical and operational support.

Implementing the NCHDs could be challenging considering resource allocation structures and the political landscape. In Ghana, financial, human, material and other resources are mainly allocated to regions and districts [43]. Thus, funding NCHDs could be problematic as they are not part of the administrative and governance structures of the GHS. However, this could be addressed by organising capacity building via the NCHD clusters and developing resource sharing mechanisms among districts within NCHDs. Furthermore, local government authorities might not support NCHD-driven programming as it does not align with electoral boundaries where they campaign for votes.

### Methodological implications for zone design and limitations

The low number of flows between many origin-destination pairs, the large number of residential communities and few hospitals affected the converging of the automated TTWA approach. The automating challenge shows that zoning applications can be contextual depending on the setting, data source and service that generated the data. Haynes and colleagues [8] observed that zone design can be challenging for rare diseases that generate no or fewer data from some parts of the study area. Whilst births are not rare, few women gave birth in many parts of the study area. Hence, the nature of our data may explain difficulties applying an existing automated tool [15].

Whilst findings are specific to attendance for childbirth, the methods in this study could be applied to other health events with patient residential and hospital addresses. Levels of self-containment for birth attendance might be similar to other events as residents are likely to use the secondary level hospitals in their NCHD rather than cross over to others. In Ghana, when seeking non-emergency care that is available at primary facilities for scabies, patients travel to hospitals in a pattern broadly comparable with the flows in Fig. 4 [44]. Thus, the NCHD zones based on hospital use patterns are likely to remain highly self-contained in the presence of shorter trips to primary health centres.

Aggregating flows from small areas into larger areas typically increases self-containment, the number of journeys starting and ending within a zone. Thus greater self-containment would be expected for flows based on NCHDs relative to MDAs, given that the former are larger zones. However, since NCHDs had greater self-containment than similar zones of comparable size, this suggests the TTWA algorithm effectively delineated groups of facilities and the populations they serve (Fig. 6).

There were manual interventions used here to make zones contiguous. Alternative methods overcome this shortfall by enforcing spatial contiguity in automated approaches [8]. Relative to aggregate data, individual data were incomplete in some hospitals. However, the correlation between aggregated and individual data has improved compared with a previous study from the same area [24]. The impact of private health facilities on the NCHD is likely to be low because there is no district served by private facilities only; birthing services are free in public facilities; and only 6% of women gave birth in private health facilities in the Eastern region [45]. Journeys to primary care facilities were not included in this study. However, they are likely to be shorter and highly localised. Therefore, primary care flows are unlikely to affect the NCHDs substantially. Future studies could use data from different years to improve record completeness and include private facilities. Since we did not incorporate attendance by Eastern region residents in neighbouring regions’ facilities, this study did not account for edge effects, the tendency for cross-boundary healthcare access to impact geographical accessibility measures [46]. A nationwide analysis could resolve or minimise the edge effect resulting from inter-regional movement.

### Transferability and future research

NCHDs could be regularly updated as movement patterns evolve, since the routine health data is continuously available to health managers. The open-source District Health Information Systems (DHIS) for routine health data management is widely implemented in many LMICs, especially across Africa [26]. The widespread DHIS presents the opportunity to scale up or replicate this study in other countries. However, the mix of public, private and faith-based healthcare providers varies across African countries [47] and private health facility integration within DHIS reporting is limited in some countries [48]. Thus, health system fragmentation in some countries, national health insurance systems influencing choice of facility, and country-specific data availability constraints could limit the replication ot this study elsewhere in Africa.

Subsequent studies should analyse routine data for other health outcomes. Our analysis shows how routine health data collected electronically via health information systems can be used for monitoring local service delivery progress [49], strengthening the case for investment in such systems [50]. Subsequent studies could also explore how the use of boundaries designed from patient flows affects health systems performance assessment systems. National systems such as Uganda’s district league table have been criticised as lacking a robust statistical basis [3], and similar methods are used in Ghana [4]. The use of more appropriate boundaries could address some of these criticisms [51]. Pregnancy and childbirth-related risk perceptions influence choice of place for antenatal care, obstetric emergencies and normal birth in Ghana [52]. Also, service availability will drive patterns as nearby primary facilities treating fevers or diarrhoea might not provide birthing services, but bypassing could be prevalent even for non-emergency cases such as scabies [44]. Therefore, the variations in patient travel for different services should be investigated in future studies as patterns for in/outpatient, emergencies/non-emergencies, communicable/non-communicable diseases, and other public health events are likely to differ, forming different zones.

## Conclusions

This study presents the first insights into applying zone design methods to maternal health birthing services using routine health data. The resultant NCHD zones were used to measure access to maternal health care. By design, most trips for birthing services start and end within NCHD, making the population denominators more stable. The TTWA algorithm had to be tailored to the available data mainly due to the number of health facilities and frequency of trips in the study area, compared with high-income country settings where the algorithm was previously used. Notwithstanding, the methodology has potential for scale-up nationally and elsewhere in Sub-Saharan Africa, particularly as investments in routine health systems continue and increasing availability of individual transactional data captured through the DHIS or other electronic health systems. This study is highly relevant to strategies by the GHS to develop health service areas.

## Data Availability

The demographic dataset analysed during the current study is openly available in the WoldPop repository, https://www.worldpop.org/ The service provision assessment dataset analysed during the current study are available from the correspondent author on reasonable requst. The birth datasets analysed during the current study are not publicly available due to confidentiality and data licencing restrictions from the Ghana Health Service. They can be obtained from the Ghana Health Service (https://www.ghs.gov.gh/contact-us) with reasonable request.

## Abbreviations

HAS: Health service area
TTWA: Travel to work areas
WHO: World Health Organisation
LMIC: Low and middle income countries
GHS: Ghana Health Service
DHIMS: District Health information Management Systems
SPA: Service provision assessment
NCHD: Natural catchment health district
MDA: Municipal and district assembly
DHIS: District Health Information Systems
